# Crizotinib Resistance Mediated by Autophagy Is Higher in the Stem-Like Cell Subset in ALK-Positive Anaplastic Large Cell Lymphoma, and This Effect Is MYC-Dependent

**DOI:** 10.3390/cancers13020181

**Published:** 2021-01-07

**Authors:** Chuquan Shang, Bardes Hassan, Moinul Haque, Yuqi Song, Jing Li, Dongzhe Liu, Eva Lipke, Will Chen, Sylvie Giuriato, Raymond Lai

**Affiliations:** 1 Department of Laboratory Medicine and Pathology, University of Alberta, Edmonton, AB T6G 2R3, Canada; chuquan@ualberta.ca (C.S.); dr_bardis@cu.edu.eg (B.H.); moinul@ualberta.ca (M.H.); yqsong20@mails.jlu.edu.cn (Y.S.); lijing2020@hrbmu.edu.cn (J.L.); sanford5529@163.com (D.L.); evalipke@gmx.de (E.L.); will.chen@ualberta.ca (W.C.); 2 Department of Pathology, Faculty of Veterinary Medicine, Cairo University, Giza 12211, Egypt; 3 Norman Bethune Health Science Center of Jilin University, Changchun 130021, China; 4 Electron Microscopy Center, Basic Medical Science College, Harbin Medical University, Harbin 150080, China; 5 Department of Hematology and Oncology, Shenzhen University General Hospital, Shenzhen 518055, China; 6 Laboratory of Biology and Chemistry, Basic Medical Science College, Harbin Medical University, Harbin 150080, China; 7 Department of Life Sciences, Albstadt-Sigmaringen University, 72488 Sigmaringen, Germany; 8 CRCT, INSERM U1037-Université Toulouse III-Paul Sabatier-CNRS ERL5294, F-31037 Toulouse, France; sylvie.giuriato@inserm.fr; 9 Department of Oncology, University of Alberta, Edmonton, AB T6G 2R3, Canada

**Keywords:** autophagy, ALK + ALCL, cancer stem-like cells, MYC, crizotinib, chloroquine, resistance

## Abstract

**Simple Summary:**

Autophagy is a cell survival and recycling mechanism which protects cancer cells upon therapeutic drug treatment. Here we investigated the impact of autophagy inhibition in a cancer of lymphoid origin, namely ALK-positive anaplastic large cell lymphoma (ALK + ALCL). We inhibited autophagy in two distinct cell subsets of ALK + ALCL, one of which we previously shown to possess more stem-like and tumorigenic properties. Our study found that blockage of autophagy in the stem-like subset resulted in marked drug-sensitization to crizotinib, a current therapeutic agent used to treat ALK + ALCL. We also found differential involvement of the *Myc*oncogene in the autophagy process within the two subsets and identified its relative importance to the stem-like population. Our research suggests inhibition of autophagy alongside crizotinib preferentially targets stem-like cells, thus improving crizotinib therapy.

**Abstract:**

Previously it was shown that autophagy contributes to crizotinib resistance in ALK-positive anaplastic large cell lymphoma (ALK + ALCL). We asked if autophagy is equally important in two distinct subsets of ALK + ALCL, namely **R**eporter **U**nresponsive (**RU**) and **R**eporter **R**esponsive (**RR**), of which RR cells display stem-like properties. Autophagic flux was assessed with a fluorescence tagged LC3 reporter and immunoblots to detect endogenous LC3 alongside chloroquine, an autophagy inhibitor. The stem-like RR cells displayed significantly higher autophagic response upon crizotinib treatment. Their exaggerated autophagic response is cytoprotective against crizotinib, as inhibition of autophagy using chloroquine or shRNA against *BECN1* or *ATG7* led to a decrease in their viability. In contrast, autophagy inhibition in RU resulted in minimal changes. Since the differential protein expression of MYC is a regulator of the RU/RR dichotomy and is higher in RR cells, we asked if MYC regulates the autophagy-mediated cytoprotective effect. Inhibition of MYC in RR cells using shRNA significantly blunted crizotinib-induced autophagic response and effectively suppressed this cytoprotective effect. In conclusion, stem-like RR cells respond with rapid and intense autophagic flux which manifests with crizotinib resistance. For the first time, we have highlighted the direct role of MYC in regulating autophagy and its associated chemoresistance phenotype in ALK + ALCL stem-like cells.

## 1. Introduction

Macroautophagy, more commonly referred to as autophagy, is a process originally discovered in yeasts as a survival mechanism in the face of starvation [1,2]. Autophagy is now known to be an important homeostatic process in human cells, in which misfolded proteins, damaged organelles and intracellular pathogens are removed through the autophagosome/lysosome pathway [3,4]. The biological relevance and importance of autophagy have been studied and established in many physiologic and pathologic states, including cancer, metabolic diseases, cardiovascular disorders, pulmonary diseases, aging, infections and neurodegenerative disorders [5,6,7,8]. In the field of cancer biology, autophagy is believed to be tumor suppressive in the early stages of tumorigenesis; however, in the later stages of tumorigenesis, autophagy can function as a tumor promoter by providing energy and nutrients for the rapidly growing cancer cells and by conferring cytoprotection against chemotherapeutic agents [9,10,11]. In keeping with its role in chemoresistance, a number of clinical trials have shown promising results regarding the use of autophagy inhibitors (such as chloroquine and its derivatives hydroxychloroquine and 3-methyladenine) in sensitizing cancer cells to chemotherapeutic agents [11,12]. In recent years, the connection between autophagy and other important aspects of cancer cell biology, such as apoptosis [2,13] and cancer stemness [14,15] have been increasingly recognized.

ALK-positive anaplastic large cell lymphoma (ALK + ALCL) is a specific type of T-cell non-Hodgkin lymphoma affecting mostly children and young adults [16,17]. These tumors are characterized by the presence of the t(2;5)(p23;q35) chromosomal aberration, leading to the generation of the fusion gene protein, NPM-ALK, which embodies the constitutively active tyrosine kinase domain [18,19]. NPM-ALK is one of the most versatile oncoproteins, as it can contribute to most of the hallmarks of cancer described [20]. While doxorubicin-based polychemotherapy has been the standard treatment for ALK + ALCL [21], crizotinib, the first FDA-approved ALK tyrosine kinase inhibitor, has been used clinically [22,23]. Nonetheless, drug resistance and disease relapse remain to be a challenge [21,24,25,26]. One of the key mechanisms for crizotinib resistance has been attributed to mutations identified at the tyrosine kinase domain of NPM-ALK [23]. Other mechanisms also exist. For example, recent reports described that inhibition of BCL2 and autophagy can enhance crizotinib-induced inhibition on ALK + ALCL cell viability, suggesting that BCL2 and the autophagy pathway contribute to crizotinib resistance in these cells [27]. Another important mechanism underlying drug resistance is related to intra-tumoral heterogeneity, which has not been extensively studied in ALK + ALCL. In this regard, our group has previously described two distinct cell populations in ALK + ALCL cell lines, namely **R**eporter **U**nresponsive (**RU**) and **R**eporter **R**esponsive (**RR**) cells, which differ from each other in their responsiveness to a Sox2 reporter [28]. In another study, we have shown that RR cells are more stem-like compared to RU cells, as they have higher levels of clonogenicity and resistance to doxorubicin [29]. Thus, it is possible that intra-tumoral heterogeneity plays a role in the context of crizotinib resistance in ALK + ALCL cells. Specifically, we hypothesize that RR cells are more resistant to crizotinib than RU cells.

In this study, we first showed that crizotinib resistance is indeed significantly higher in RR cells, which represent the small subset of stem-like cells in ALK + ALCL. In view of the previous study that autophagy contributes to crizotinib resistance in ALK + ALCL [30], we asked if the higher level of crizotinib resistance in RR cells is due to the fact that they can express a significantly higher autophagic response compared to RU cells. Lastly, since we previously found that MYC is a key regulator of the RU/RR dichotomy, with MYC being more highly expressed in RR cells [29,31,32], we assessed if experimental manipulation of the MYC expression level can significantly modulate the autophagy response in ALK + ALCL, and thus their sensitivity to crizotinib.

## 2. Results

### 2.1. Generation of LC3-Transfected RR and RU Cells

**Figure 1:**
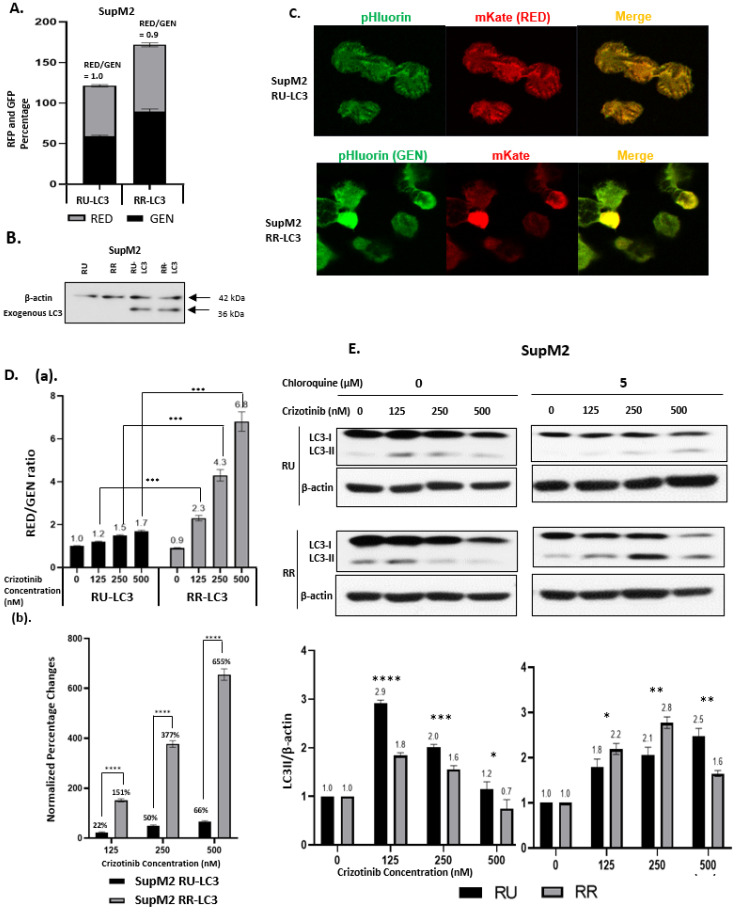
Crizotinib-induced autophagy activity is significantly enhanced in Reporter Responsive (RR) cells than Reporter Unresponsive (RU) cells. (**A**) RED/GEN ratio in LC3 transduced SupM2 RU and RR subset cells. SupM2 RU cells were used as the baseline of GEN and RED. (**B**) Western blotting analysis to confirm the transduction of exogenous LC3 in SupM2 RU and RR cells. The bands at 36 kDa were exogenous LC3 fused with fluorescent probes. (**C**) Confocal microscopy was performed to verify the expression of exogenous LC3. RU-LC3 and RR-LC3 at the steady state showed the co-expression of both red and green fluorescence signals; cells were visualized with phase contrast. (**D**) (**a**) RED/GEN ratio in SupM2 RU-LC3 and RR-LC3 cells following treatment with 0, 125, 250, and 500 nM of crizotinib. Cells were collected for flow cytometry measurement after 24 h. (**b**) Data provided in A is expressed as percentages of change for each crizotinib dosage. Data normalized to control dimethyl sulfoxide (DMSO/Crizotinib at 0 nM) group, respectively. (**E**) Changes in LC3 protein level in response to crizotinib are different between RU and RR cells. Western blot analysis of SupM2 RU and RR cells treated with different dosages of crizotinib (0, 125, 250, and 500 nM) with or without 5 µM of chloroquine for 24 h. Original blots are shown in Figure S4. ImageJ Software was used to measure bands intensity. Three independent experiments were performed. Data shown as mean ± standard deviation (SD), *n* = 3, * *p* < 0.05, ** *p* < 0.01, *** *p* < 0.001, **** *p* < 0.0001, Student’s *t* test.

The generation of RU and RR cell subsets derived from ALK + ALCL cell lines have been detailed previously [28]. Briefly, we transduced ALK + ALCL cell lines with a commercially available Sox2 regulatory factor-2 (SRR2) reporter, which expresses GFP or luciferase in proportion to the level of Sox2 transcription activity. Stably transduced cell lines were then subjected to flow cytometric sorting, and RU and RR cells were purified based on their differential GFP expression. To compare the autophagic activity between RU and RR cells, we generated pHluorin-mKate2-tagged LC3 transfectants for these two cells’ subsets derived from SupM2 and Karpas 299. The characteristics of RU and RR cells have been detailed in our previous publications [28,33]. We use GEN to specifically stand for the green signal (B530) from pHluorin, a pH-sensitive GFP variant, and RED to stand for the red signal (B695) from the next generation of far-red fluorescent protein TagFP635 (mKate), which is pH-resistant. As shown in Figure 1A, the RED/GEN ratios for RU-LC3 and RR-LC3 at the steady state were not significantly different (1.0 versus 0.9) (*p* > 0.05). Furthermore, the exogenous LC3 was confirmed by western blotting, and equal protein expression was identified in both subsets (Figure 1B). Triplicate experiments were performed, and results of a representative run are illustrated. When we performed confocal microscopy, the expression of GEN and RED was readily detectable in these stably transfected RU-LC3 cells and RR-LC3 cells (Figure 1C). Of note, despite that RR cells are known to have a baseline level of GFP expression due to their intrinsic SOX2 reporter activity [32,34], the green fluorescence from pHluorin has been shown to be more sensitive to pH decreases as a result of autophagy in comparison to the green fluorescence from GFP [33]. Thus, it appears that the intrinsic GFP expression in RR cells was overshadowed by the GEN signal coming from the LC3 plasmid.

### 2.2. Crizotinib-Induced Autophagy Is Significantly Enhanced in RR Cells

It has been published that tyrosine kinase inhibitor, crizotinib, can trigger autophagy in ALK + ALCL cells [27,30]. Thus, we asked if the crizotinib-triggered autophagy response is different between RU and RR cells. As illustrated in Figure 1D(a), the RED/GEN ratio in RU cells increased in response to crizotinib in a dose-dependent manner, being 1.2 at 125 nM, 1.5 at 250 nM and 1.7 at 500 nM. In comparison, RR cells showed significantly higher crizotinib-induced increases in the RED/GEN ratio, being 2.3 at 125 nM, 4.3 at 250 nM and 6.8 at 500 nM. The differences between RU and RR cells at these three crizotinib dosages are statistically significant (*p* < 0.001). To highlight these differences, we summarized the percentages of change in RED/GEN (compared to DMSO-treated groups) for each crizotinib dosage based on the following formula:

$$Normalized\;Percentage\;Changes=\frac{{\left(\frac{RED}{GEN}\right)}_{CZ}-{\left(\frac{RED}{GEN}\right)}_{DMSO}}{{\left(\frac{RED}{GEN}\right)}_{DMSO}}\times 100\%$$

As shown in Figure 1D(b), the differences are 22% versus 151% (125 nM), 50% versus 377% (250 nM) and 66% versus 655% (500 nM). Similar experiments were performed using Karpas 299. Although Karpas 299 appeared to have higher IC_50_ than SupM2 cells, we observed the same trends (Figure S1) by using higher dosages of crizotinib.

We then performed western blot studies and the results are illustrated in Figure 1E. In the absence of chloroquine, both RU and RR cells displayed a substantial upregulation of LC3II at a relatively low dose of Crizotinib (125 nM). With an increase in the crizotinib dosage to 250 and 500 nM, the LC3II level gradually decreased in both RU and RR cells. In the presence of autophagy inhibition (i.e., chloroquine at 5 µM), RU cells showed a crizotinib dose-dependent accumulation of LC3II, supporting that autophagy was effectively inhibited. At a crizotinib dose of ≤250 nM, RR cells also showed a crizotinib dose-dependent increase in LC3II, although the LC3II accumulation was more profound compared to that of RU cells (*p* < 0.05). At a dose of 500 nM of Crizotinib, the LC3II level decreased in RR cells. We believe that this decrease in LC3II in RR cells at 500 nM of crizotinib is likely due to the insufficient inhibition of autophagy by 5 µM of chloroquine.

**Figure 2:**
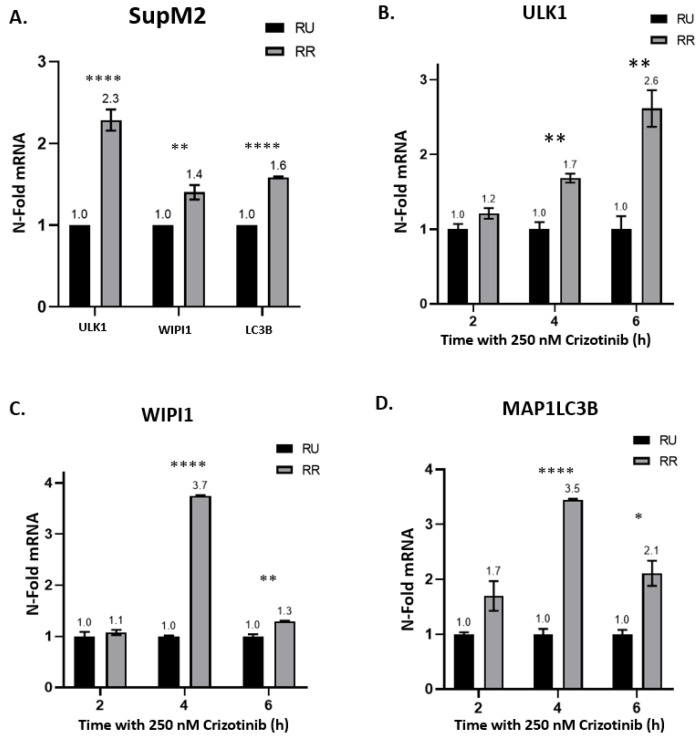
Autophagy related genes highly expressed in SupM2 RR cells compared to RU cells at mRNA level. (**A**) The relative mRNA expression of ULK1, WIPI1, and MAP1LC3B in SupM2 RU and RR cells at steady state. Results were detected by quantitative PCR between SupM2 RU and RR cells. (**B**–**D**) The relative mRNA expression of ULK1, WIPI1, and MAP1LC3B in SupM2 RU and RR cells treated with 250 nM of crizotinib. Cells were collected 2 h, 4 h, and 6 h after drug treatment. Data normalized to SupM2 RU cells and shown as mean ± standard error of mean (SEM), *n* = 3; * *p* < 0.05, ** *p* < 0.01, **** *p* < 0.0001, Student’s *t* test. Three independent experiments were performed.

We then compared the mRNA expression of several key autophagy-related genes between RU and RR cells. The three genes chosen were (1) Unc-51 like Autophagy Activating Kinase 1 (ULK1), which is one of the key factors for the autophagic initiation complex [35,36]; (2) WD Repeat Domain, Phosphoinositide Interacting 1 (WIPI1), which drives phagophore elongation at an early stage [37,38]; and (3) Microtubule Associated Protein 1 Light Chain 3 Beta (MAP1LC3B), which is one of the autophagosome markers at the late stage of elongation [39,40,41]. As shown in Figure 2A, RR cells had significant higher expression of all three genes compared to RU cells at the steady state. More specifically, RR cells expressed 2.3-fold more ULK1 (*p* < 0.0001), 1.4-fold more WIPI1 (*p* < 0.01) and 1.6-fold more MAP1LC3B (*p* < 0.0001) than RU cells. When treated with 250 nM of crizotinib, RR cells showed significantly higher upregulation of these three genes than RU cells (Figure 2B–D) after 4 and 6 h of treatment. Similar experiments were performed in Karpas 299 cells, and, as shown in Figure S2, similar trends were observed. Indeed, Karpas 299 RR cells had higher basal ULK1, WIPI1 and MAP1LC3B mRNA levels compared to Karpas 299 RU cells. Moreover, crizotinib treatment was consistently associated with upregulated expression of these three genes, in comparison to RU cells. Of note, Karpas RR cells showed significantly higher expression of ULK1 and WIPI1 after 2 h of 500 nM crizotinib treatment. These findings correlate with the more rapid and intense crizotinib-induced autophagy flux in RR cells.

### 2.3. The Autophagy Response in RR Cells Contributes to Crizotinib Resistance

**Figure 3:**
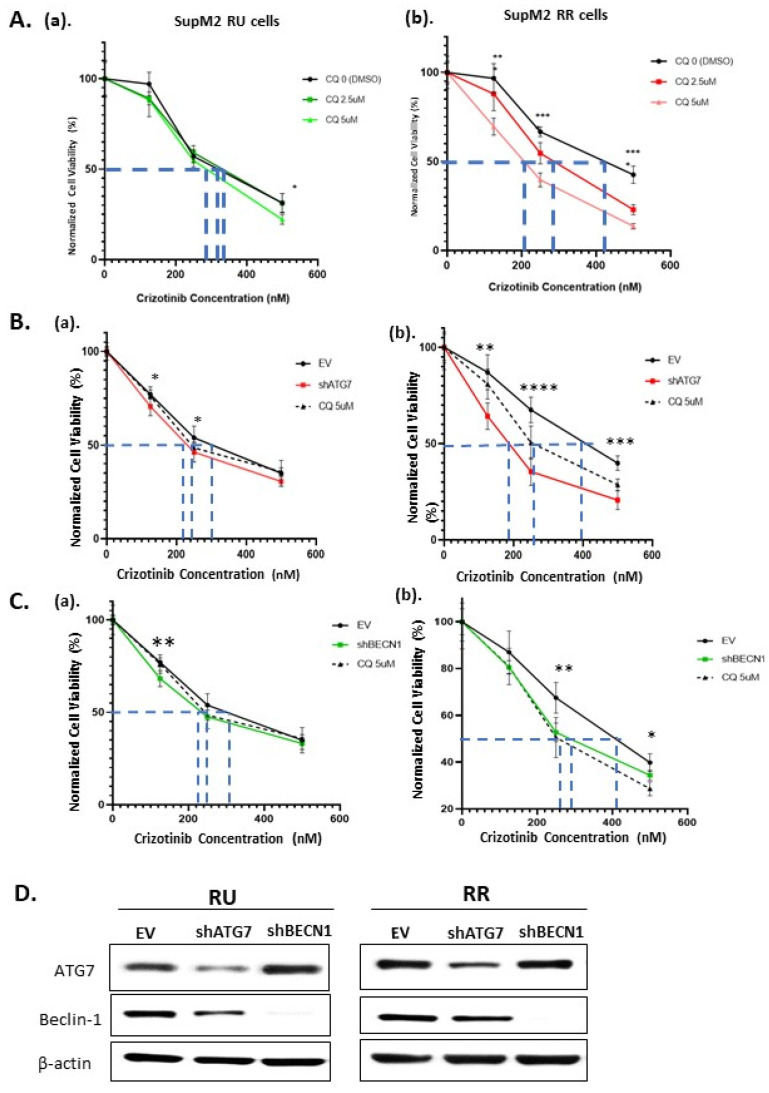
Inhibition of autophagy significantly enhanced the crizotinib-induced loss of cell viability in RR but not RU cells. (**A**) Cell viabilities of SupM2 RU and RR cells treated with different dosages of crizotinib (0, 125, 250, and 500 nM) and chloroquine (0, 2.5, 5 µM). Cell viability was determined by Trypan Blue after 24 h. (**B**) SupM2 RU (**a**) and SupM2 RR (**b**) cells were treated with different dosages of crizotinib (0, 125, 250, 500 nM) for 24 h following knockdown of ATG7 or administration of 5 µM chloroquine (positive control). (**C**) SupM2 RU (**a**) and SupM2 RR (**b**) cells were treated with different dosages of crizotinib (0, 125, 250, 500 nM) for 24 h following knockdown of Beclin-1 or administration of 5 µM chloroquine (positive control). Original blots are shown in Figure S4. Data shown as mean ± standard deviation (SD), *n* = 3, * *p* < 0.05, ** *p* < 0.01, *** *p* < 0.001, **** *p* < 0.0001, Student’s *t* test. Three independent experiments were performed. (**D**) Western blots to assess the efficiency of shRNA knockdown of ATG7 or Beclin-1 in both cells. The most representative blot was chosen.

Since autophagy has been previously shown to provide cytoprotective effect against crizotinib in ALK + ALCL cells [27,30], we asked if the high autophagy capacity in RR indeed translates into a significantly higher cytoprotective effect against crizotinib. As shown in Figure 3A, RR cells had significantly higher IC_50_ of crizotinib compared to RU cells (409 nM vs. 326 nM) (*p* < 0.05). These IC_50_ results are consistent with that for SupM2 published in the literature. Specifically, the IC_50_ for SupM2 is 387 nM of crizotinib in the reference of the Genomics of Drug Sensitivity in Cancer (GDSC) database [42], and this dosage lies between those for RR and RU cells.

With the addition of chloroquine, there was no significant change in the IC_50_ in RU cells. In contrast, there was a significant decrease in the IC_50_ in RR cells, dropping from 409 nM to 281 nM at 2.5 µM of chloroquine and 196 nM at 5 µM of chloroquine. These results suggest that autophagy contributes to crizotinib resistance in RR but not RU cells. We repeated the same experiment in Karpas 299 cells. Of note, although Karpas 299 cells had higher IC_50_ of chloroquine compared to SupM2 cells, similar trends were observed, as shown in Figure S3.

### 2.4. The Loss of Cell Viability in RR Cells Is Due to Autophagy Inhibition

To substantiate that the loss of cell viability was indeed due to autophagy, we repeated the experiments described in Section 3 by silencing, using shRNAs, two specific autophagy regulators, namely BECN1 (which encodes Beclin-1 protein) and Autophagy Related 7 (ATG7), both of which are involved in the regulation of autophagy [43]. Interestingly, we found that the knockdown of either ATG7 (Figure 3B) or Beclin-1 (Figure 3C) significantly decreased the IC_50_ of crizotinib in RR cells, but no substantial effect was found in RU cells. In other words, these shRNA species induced the same effects as chloroquine, as illustrated in Figure 3. Using western blots, we confirmed that both Beclin-1 and ATG7 were successfully downregulated with their respective shRNA species (Figure 3D).

### 2.5. Higher MYC Expression Enhances Crizotinib Chemoresistance via Autophagy in RR Cells

**Figure 4:**
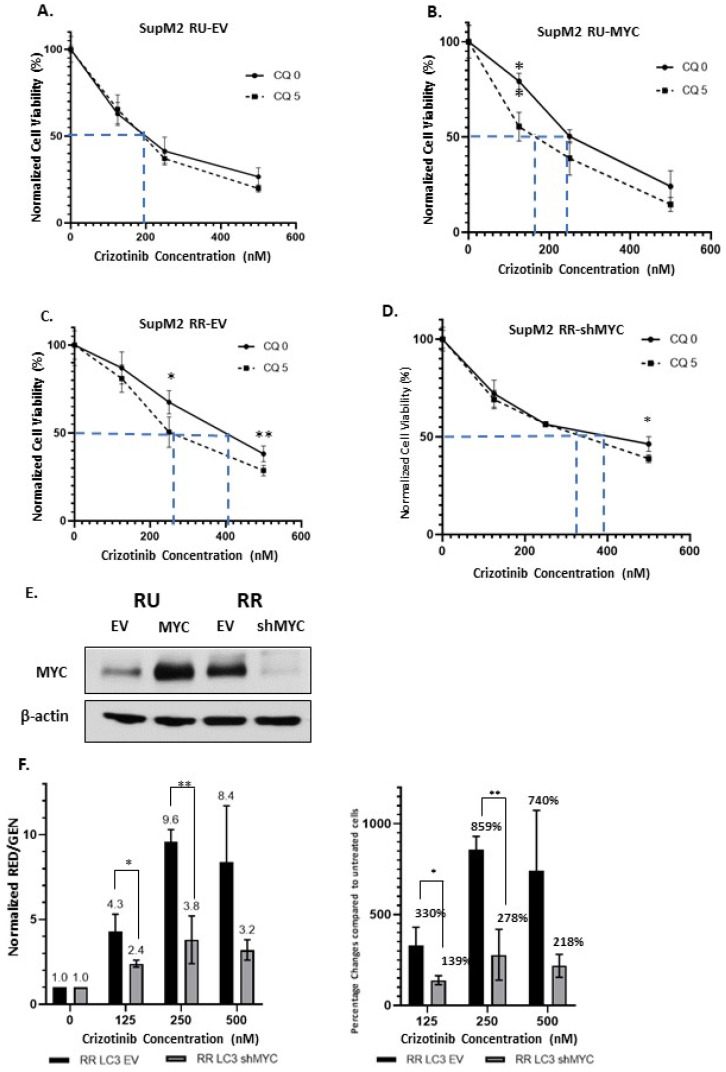
MYC regulates autophagy in ALK-positive anaplastic large cell lymphoma (ALK + ALCL) cells treated with crizotinib. (**A**–**D**) Cell viability after treating with different dosages of crizotinib (0, 125, 250, 500 nM) for 24 h following the modulation of MYC. The MYC protein expression level was modulated by using a MYC overexpression plasmid (into RU cells, **A**,**B**) or a shRNA against MYC (into RR cells, **C**,**D**). (**E**) Western blot to confirm the expression of MYC in SupM2 RU and RR cells. (**F**) The RED/GEN ratio and the normalized percentage changes with crizotinib treatment (0, 125, 250, 500 nM) for 24 h after knockdown of MYC by shRNA in SupM2 RR-LC3 cells. Original blots are shown in Figure S4. Data from three independent experiments and shown as mean ± standard deviation (SD), *n* = 3, * *p* < 0.05, ** *p* < 0.01, Student’s *t* test.

We have previously shown that MYC is a key driver of the RU/RR dichotomy, with a high MYC expression level correlating with the RR phenotype [32]. Thus, we asked if the protein level of MYC can regulate crizotinib-induced autophagy in ALK + ALCL cells. As shown in Figure 4A–D, in MYC-transfected RU cells (Figure 4A,B), autophagy inhibition by chloroquine sensitized ALK + ALCL cells to crizotinib. By comparison, shRNA downregulation of MYC in RR cells resulted in the abrogation of the sensitizing effect of chloroquine (Figure 4C,D), although residual effect remained to be detected at a crizotinib dosage of 500 nM. In other words, MYC-transfected RU cells behaved similarly to “native” RR cells, whereas RR cells subjected to MYC knockdown behaved similarly to “native” RU cells. The efficiency of MYC overexpression in RU cells and MYC knockdown in RR cells is illustrated in Figure 4E.

To assess how our experimental manipulation of the MYC protein level directly modulates the autophagic flux, we subjected RR-LC3 cells derived from SupM2 to knockdown MYC by shRNA. Results are illustrated in Figure 4F. Compared to RR-LC3 transfected with an empty vector (EV), RR-LC3 cells transfected with MYC shRNA showed significantly less upregulation in the RED/GEN ratios with increasing dosages of crizotinib at 125 nM and 250 nM, but marginally outside the statistical significance at 500 nM. These results again support the concept that RR cells with MYC knockdown adopted the phenotype of RU cells with respect to the autophagic response induced by crizotinib.

## 3. Discussion

There is accumulating evidence that autophagy contributes to chemoresistance in cancer cells [11,12,30]. In the context of ALK + ALCL and crizotinib, Mitou et al. have shown that crizotinib upregulates autophagy in these lymphoma cells [30]. Importantly, they found that autophagy inhibitors (i.e., chloroquine) can effectively enhance crizotinib-induced anti-tumoral action in ALK + ALCL cells, and that the combination of autophagy inhibitors and tyrosine kinase inhibitors may improve the therapeutic efficacy for these patients. Results from this current study have confirmed the observation that crizotinib activates autophagy in ALK + ALCL cells. Furthermore, we have shown that the crizotinib-induced autophagy is significantly enhanced in RR cells, the cancer stem-like cell subset. Correlating with the concept that autophagy can contribute to chemoresistance, we found a substantial difference in the crizotinib sensitivity between RU and RR cells, with the IC_50_ for RU being significantly lower than that of RR cells (326 nM versus 409 nM of crizotinib). Since similar results were obtained with Karpas 299, our conclusion may be generalized to ALK + ALCL cells. Overall, our findings echo those of Mitou et al., and they are in support of the concept of combining autophagy inhibitors and tyrosine kinase inhibitors in treating ALK + ALCL patients, although the current study has highlighted the importance of this therapeutic strategy in eliminating the cancer stem-like cell population.

For the first time, the importance of MYC in regulating crizotinib-induced autophagy and crizotinib resistance in ALK + ALCL cells is revealed. Thus, shRNA downregulation of MYC in RR cells significantly weakened autophagic response and its associated cytoprotective effect against crizotinib, whereas overexpression of MYC in RU cells resulted in the opposite effects. A search of the literature has revealed only a handful of publications focusing on the functional connection between MYC and autophagy. In 2003, Tsuneoka et al. demonstrated that the overexpression of MYC in rat fibroblasts significantly increased the number of autophagic vacuoles [44]. In a more recent study, it was found that the loss of MYC can reduce autophagy and further restrain cellular proliferation in osteosarcoma cells [45]. Thus, results from these two studies support the concept that MYC can regulate autophagy. Exactly how MYC regulates autophagy in ALK + ALCL cells requires further studies. In the literature, it was reported that suppression of MYC can inhibit autophagy by reducing c-Jun N-terminal kinase (JNK1) and Bcl2 [46]. Other recent studies highlight different MYC/microRNA/Autophagy axes that are involved in cancer chemoresistance [47,48]. The relevance of these mechanisms in ALK + ALCL needs to be examined.

Our observation that RR cells, being stem-like, possess a significantly stronger autophagic response than RU cells is in alignment with their important biological roles in normal stem cells and cancer stem cells [49]. For instance, studies using autophagy-deficient mice have provided evidence that autophagy is dispensable for embryogenesis [50,51]. In normal hematopoietic stem cells, autophagy has been found to play a key role in self-preservation during the prolonged periods of quiescence by building robust repair mechanisms [52]. In CML stem cells, the inhibition of autophagy was found to improve the efficiency of the BCR-ABL tyrosine kinase inhibitor imatinib [53,54,55]. Similar to our RR cells, mammosphere cells established from human breast cancer cell lines are known to have stronger autophagic response compared to their adherent counterparts [56]. In fact, inhibition of autophagy using chloroquine can effectively suppress their ability to form spheroids and xenograft tumor formation [57]. In the context of cancer cell plasticity, it has been shown that autophagy facilitates the hypoxia-induced conversion of stem-like cells in pancreatic cancer cell lines [58]. Of note, our group has previously shown that RU cells can be induced to convert into RR cells upon oxidative stress [32]. It would be of interest to assess if inhibition of autophagy can also effectively block this conversion. If this turns out to be the case, autophagy inhibition may indeed carry the therapeutic potential of decreasing the frequency of cancer stem-like cells in tumors, and ultimately, lowering the likelihood of cancer relapse.

While the concept that autophagy plays an important role toward chemoresistance in cancer cells is well established, clinical trials combining autophagy inhibitors with conventional chemotherapeutic agents have not met the success one might hope for. As pointed by Wang et al. [59], one of the reasons for this suboptimal response may be related to lack of full appreciation of the importance and relevance of intra-tumoral heterogeneity in the context of autophagy-mediated chemoresistance. Based on the current concepts, a key mechanism in generating intra-tumoral heterogeneity is cancer cell plasticity [60]. The efficacy of autophagy inhibitors is expected to be highly dependent on the level of microenvironment stresses as well as the status of cancer cell plasticity. In situ evaluation of the expression of autophagy-related proteins in tumors may provide useful information to guide therapies targeting autophagy [61]. Using immunohistochemistry, Espina et al. examined breast comedo-ductal carcinoma in situ and found that BECN1 (Beclin-1) to be upregulated at the viable rim of intraductal cells within the hypoxic ductal niche [57]. This type of assay, especially done on treatment-unresponsive tumors, may be highly useful in assessing the mechanism of resistance.

## 4. Materials and Methods

### 4.1. Culture of Cell Lines

Two ALK + ALCL cell lines (SupM2 and Karpas 299) were cultured in Roswell Park Memorial Institute (RPMI) 1640 media (Gibco, Waltham, MA, USA). Lenti-X 293T cells were maintained in high glucose Dulbecco’s modified Eagle’s medium (DMEM, Gibco). All media were supplemented with 10% fetal bovine serum (FBS, Gibco) and 1% penicillin and streptomycin (Gibco). All cells were maintained in a 5% CO_2_ atmosphere at 37 °C. RU and RR cells and their LC3-transfectants derived from SupM2 and Karpas 299 were purified with the use of BD FACSAriaTM III, a flow cytometric cell sorter, from Becton Dickinson Bioscience, part of Becton, Dickinson and Company (BD Bioscience, Franklin Lakes, NJ, USA). After purification, cells were cultured in the presence of 0.5 µg/mL puromycin (Gibco) at all times. Details of the method have been described previously [28].

### 4.2. Cell Viability Assays

3-(4,5-dimethylthiazol-2-yl)-5-(3-carboxymethoxyphenyl)-2-(4-sulfophenyl)-2H-tetrazolium, inner salt (MTS) assay (Promega, Madison, WI, USA) and Trypan blue (Gibco) were used to measure and verify cell viability following drug treatments under the guidance of the manufacturer’s protocol. IC_50_ was calculated by GraphPad Prism software (GraphPad Software, San Diego, CA, USA).

### 4.3. Quantitative Real-Time Polymerase Chain Reaction (qRT-PCR)

RNA was extracted from cell lines with the RNeasy Plus Mini Kit (Qiagen, Valencia, CA, USA). On column DNA digestion with DNAse I (Invitrogen, Carlsbad, CA, USA) was performed to remove trace DNA. RNA concentration was measured using the spectrophotometer (Beckerman Coulter, Brea, CA, USA). Reverse Transcription (RT) reactions were performed with 1 μg of RNA using the High-Capacity cDNA Reverse Transcription Kit (Invitrogen) to build cDNA. Quantitative real-time PCR (qPCR) reactions were performed in 96-well plates (Axygen, San Francisco, CA, USA) with the Power SYBR™ Green Master Mix (Thermo Fisher Scientific, Waltham, MA, USA) according to the manufacturers’ protocol. The Mastercycler^®^ ep Realplex system (Eppendorf, Hamburg, Germany) was used for monitoring of the qPCR. The sequence of primers was:

ULK1 F-5-CCTCGCCAAGTCTCAGACGC-3 & R-5-CCCCACCGTTGCAGTACTCC-3 MAP1LC3B: F-5- AAGCAGCGCCGCACCTTCGA-3 & R-5-CGCTGACCATGCTGTGTCCG-3 WIPl1: F-5-GAGCGCCTCTTCTCCAGCAG-3 & R-5-CAGCCTTTGCCGGTTCAGCC-3 GAPDH: F-5-GGTCTCCTCTGACTTCAACAGCG-3 & R-5-ACCACCCTGTTGCTGTAGCCAA-3.

qPCR cycling was set up as denaturation at 95 °C for 2 min, followed by 50 cycles at 95 °C for 15 s, annealing at 55 °C for 15 s, and extension at 68 °C for 20 s. The relative gene expression was determined using the ΔΔ-CT method. Normalization of target gene expression was made to GAPDH and then to the experimental control group.

### 4.4. Vectors and Plasmids

Short hairpin RNA (shRNA) plasmids for MYC (TRCN0000174055), ATG7 (TRCN0000007587) and BECN1 (TRCN0000033549) were purchased from Sigma-Aldrich (Sigma Aldrich, St. Louis, MO, USA). Vectors used for overexpressing MYC (pCDH-puro-cMYC, plasmid #46970) were from Addgene (Watertown, MA, USA). The pHluorin-mKate2-tagged human LC3 plasmid (FUGW-PK-hLC3, plasmid #61460) was purchased from Addgene, and its characteristics have been previously published [33].

### 4.5. Lentiviral Based Transduction

Lenti-X 293T cells were seeded in a 100mm dish then transfected with 9 µg transfer vector, 9 µg psPax2, and 3 µg pMD2.G plasmids (Addgene) using 30 µL Lipofectamine 2000 (Thermo Fisher Scientific). Forty-eight hours after the transfection, the viral supernatant was collected for transduction, fresh DMEM was added to produce more viral supernatant after another 24 h. ALK + ALCL cells (1 × 10^6^) were incubated with 1 mL of viral supernatant containing 0.8 µg/mL polybrene (Sigma-Aldrich) for each well of a six-well plate. The plate was then spun at 1000× *g* at 32 °C for 2 h to facilitate viral infection. Following the spin-infection step, 2 mL of fresh medium was added to each well. To increase the transduction efficiency, the spin-infection step was repeated the next day with the fresh aliquot of virus supernatant. Twenty-four hours after the second spin-infection step, cells were washed with cold PBS and resuspended in fresh medium in T25 flasks (Thermo Fisher Scientific) for incubation. After 24 h, cells were then collected for subsequent assays and western blot analysis.

### 4.6. Antibodies and Chemicals

Primary antibodies used in this study included anti-MYC (Y69, 1:2000, ab32072) which was obtained from Abcam (Cambridge, MA, USA). Anti-β-actin (1:2000, sc-47778) was purchased from Santa Cruz Biotechnology (Santa Cruz, CA, USA). Anti-LC3B antibody was from Sigma-Aldrich (1:2000, Lot# 048M4810V). Beclin-1 (1:4000, #3738) and ATG7 (1:1000, #8558) antibodies were purchased from Cell Signaling (Danvers, MA, USA). Secondary antibodies used were HRP-conjugated anti-mouse (1:2000, #7076) and anti-rabbit (1:2000, #7074) (Cell Signaling). Crizotinib (PZ0191-5MG) and chloroquine (C6628-25G) were purchased from Sigma-Aldrich. All treatments were performed according to the manufacturer instructions. Stock solutions of crizotinib (1 mM) were prepared in dimethyl sulfoxide (DMSO, Sigma-Aldrich) and stock solution of chloroquine (50 mM) was prepared in water. They were then diluted into the desired concentration for further drug treatment.

### 4.7. Western Blot

Cell lysates were prepared and lysed with 1× RIPA buffer (Millipore, Burlington, MA, USA) with 0.05% protease inhibitor cocktail (Millipore) and 0.05% phosphatase inhibitor cocktail (Millipore). Protein concentrations were measured using a Pierce™ BCA Protein Assay Kit (Thermo Fisher Scientific). Cell lysates treated with SDS were subjected to SDS-PAGE and transferred onto a nitrocellulose membrane. The membrane was then blocked with 5% skim milk dissolved in TBS-Tween20 (0.1%, *v*/*v*) for 30 min. The membrane was shaken in primary antibodies for overnight in 4 °C cold room. These antibodies were probed with anti-rabbit or anti-mouse IgG conjugated with horseradish peroxidase (1:1000, Cell Signaling). The membrane was washed three times with TBS-T after secondary antibody treatment. The bands on the membrane were visualized with Pierce™ ECL western blotting substrate (Thermo Fisher Scientific) and exposed to autoradiography films (Labscientific, Highlands, NJ, USA). The intensity of bands was quantified using ImageJ Software (NIH and Laboratory for Optical and Computational Instrumentation, LOCI, University of Wisconsin, Madison, WI, USA). The ratio of LC3-II/β-actin was normalized to LC3-II/ β-actin in the control (DMSO) group, which was set to 1.

### 4.8. Flow Cytometric Quantification of Autophagic Flux

Autophagic flux was assessed on a stable clonal population of FUGW-PK-hLC3-transduced SupM2 and Karpas 299 cells, generated from our laboratory. Flow cytometry was performed using the BD LSRFortessa X-20 SORP from Becton, Dickinson & Company. In this study, we use GEN to specifically stand for the green signal (B530) from pHluorin, a pH-sensitive GFP variant, and RED to stand for the red signal (B695) from the next generation of far-red fluorescent protein TagFP635 (mKate), which is pH-resistant. During the maturation of autophagosomes, the green fluorescence decreases due to the acidic environment while the red fluorescence is still stable, thus the ratio RED/GEN can be calculated as a read-out for autophagy flux measurement. All data were analyzed and verified with software FlowJo (Becton, Dickinson & Company).

### 4.9. Statistical Analysis

Numerical data have been expressed as mean ± deviation (SD) or mean ± standard error of the mean (SEM) obtained from the number of replicates mentioned in the figure legends. The two-tailed Student’s *t* test was used to determine the significance with α = 0.05. Statistical analysis was performed with GraphPad Prism (GraphPad Software). At least three independent experiments were performed.

## 5. Conclusions

In conclusion, our findings support that the stem-like RR cells carry a significantly higher rapidity and intensity in response to crizotinib-induced autophagy than RU cells. This property contributes to the higher crizotinib resistance seen in RR cells. The findings from this study, for the first time, highlighted the direct role of MYC in regulating autophagy and its associated chemoresistance phenotype in ALK + ALCL cells. Suppression of MYC or other targets in the autophagy pathway might be useful in improving the therapeutic efficacy of crizotinib against ALK + ALCL, especially the stem-like cell population.

## Data Availability

The data presented in this study are openly available at http://doi.org/10.3390/cancers13020181.

## Supplementary Materials

The following are available online at https://www.mdpi.com/2072-6694/13/2/181/s1, Figure S1: Crizotinib-induced autophagy is significantly more enhanced in Karpas 299 RR cells than RU cells, Figure S2: Autophagy related genes are more highly expressed in Karpas 299 RR cells than RU at mRNA level, Figure S3: Autophagy inhibition using chloroquine enhances the crizotinib-induced loss of cell viability in Karpas 299 RR but not RU cells. Figure S4: Original western blots of Figure 1, Figure 3 and Figure 4.
