# Supplementary material for: Crizotinib Resistance Mediated by Autophagy Is Higher in the Stem-Like Cell Subset in ALK-Positive Anaplastic Large Cell Lymphoma, and This Effect Is MYC-Dependent
Source: Cancers (Basel). 2021 Jan 7;13(2):181. doi: 10.3390/cancers13020181 (PMC7825760; doi:10.3390/cancers13020181)
Supplement: Supplementary file 1 [file cancers-13-00181-s001.pdf]

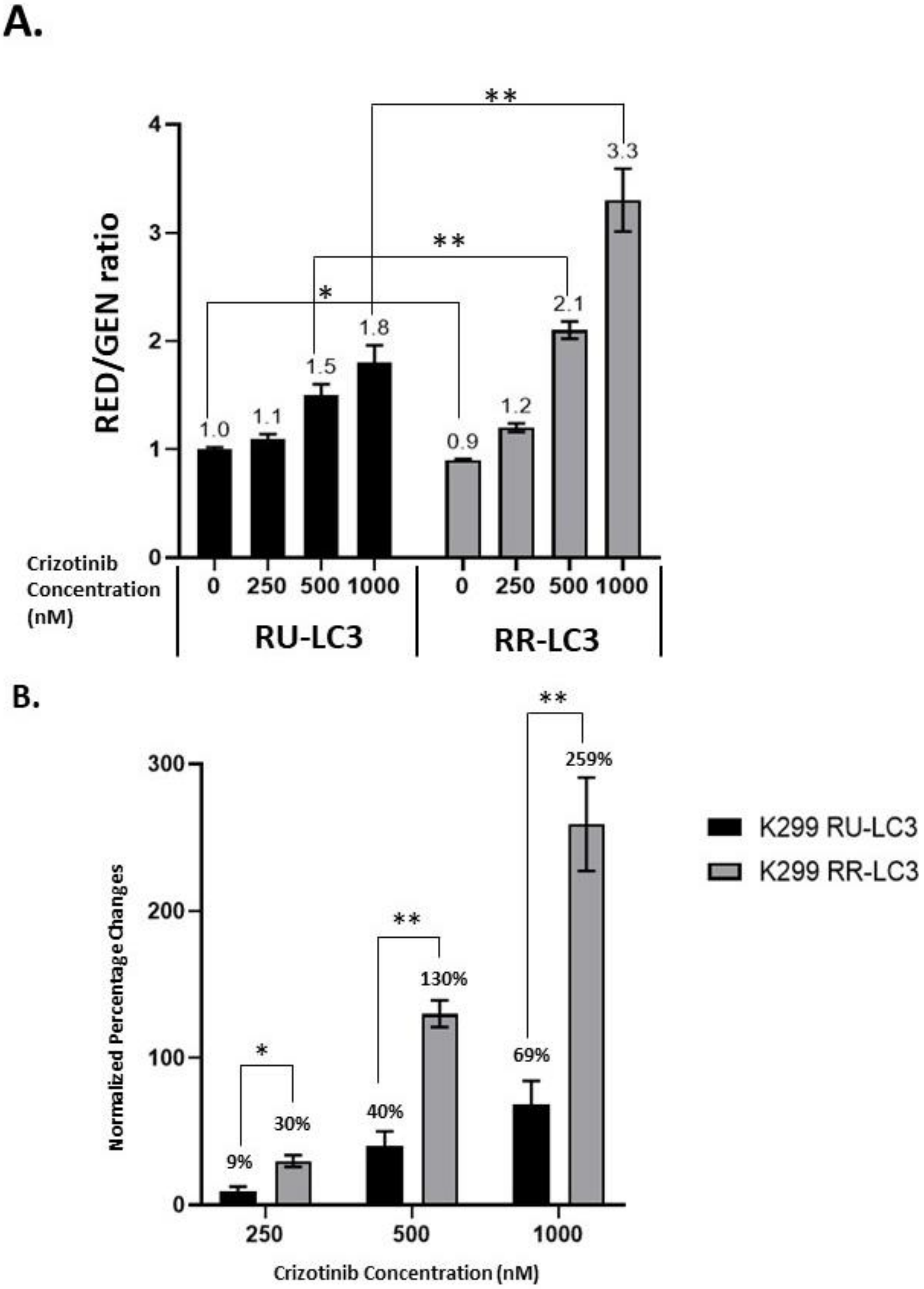

Figure S1. Crizotinib-induced autophagy is significantly more enhanced in Karpas 299 RR cells than RU cells.

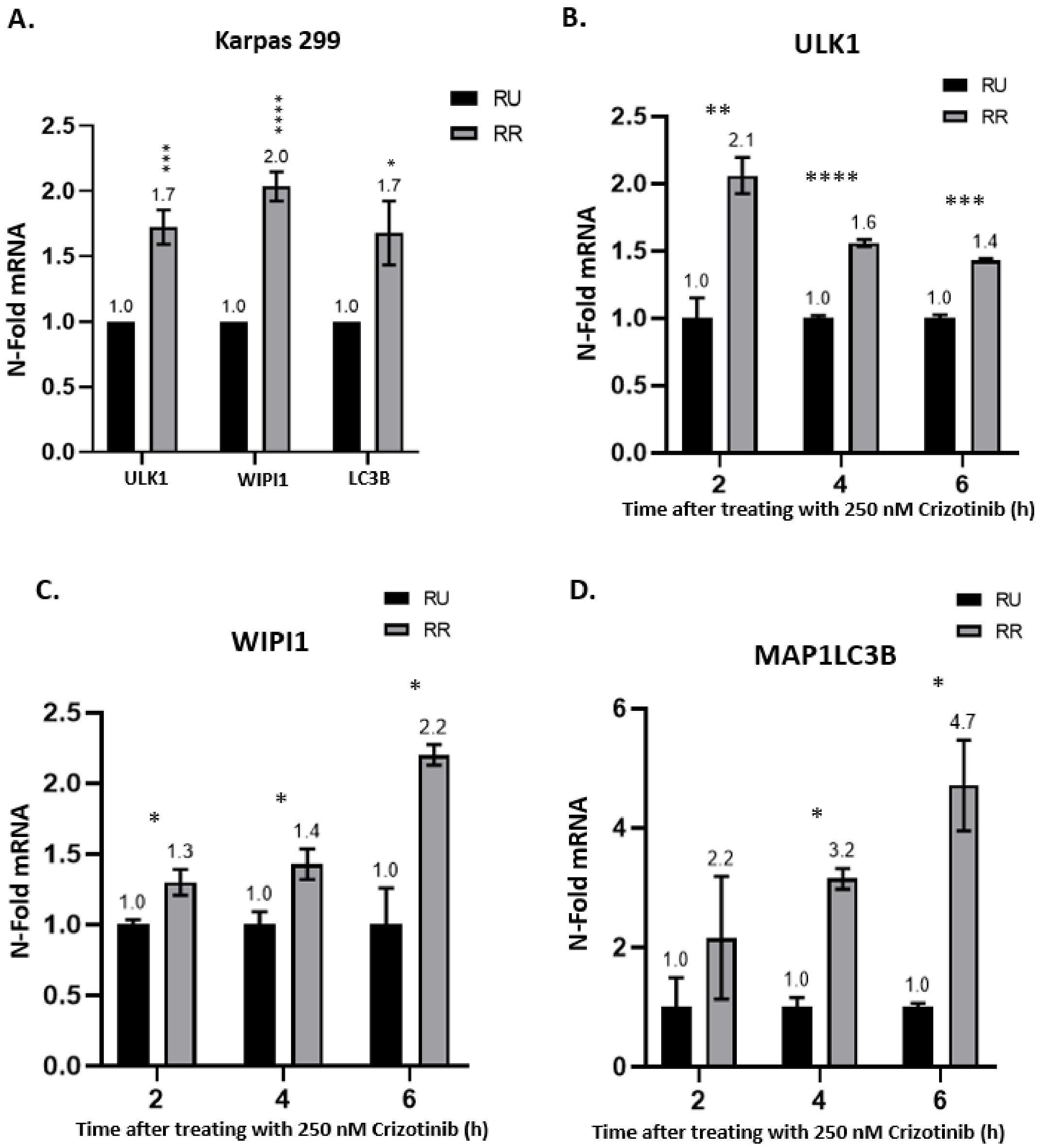

Figure S2. Autophagy related genes have higher expression in Karpas 299 RR cells than RU at mRNA level. * $p < 0.05$, ** $p < 0.01$, *** $p < 0.001$, **** $p < 0.0001$, Student's t test.

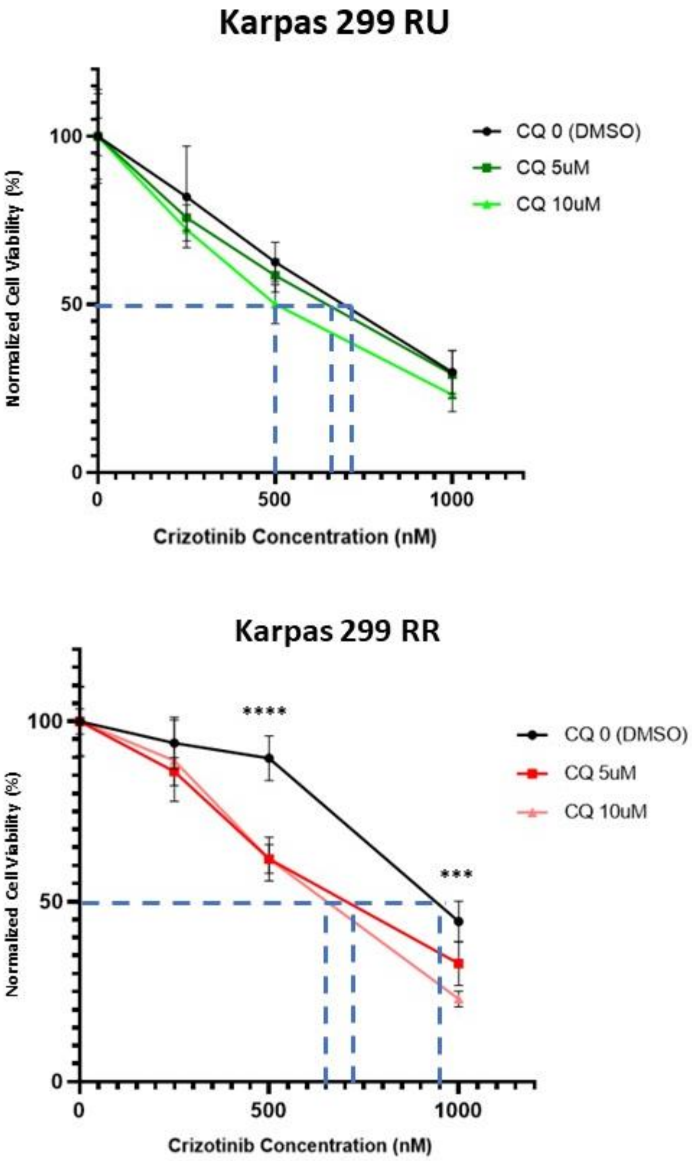

Figure S3. Autophagy inhibition using chloroquine enhances the crizotinib-induced loss of cell viability in Karpas 299 RR but not RU cells. *** $p < 0.001$, **** $p < 0.0001$, Student's *t* test.

Figure 1B

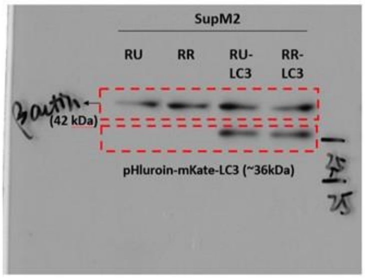

Figure 1E

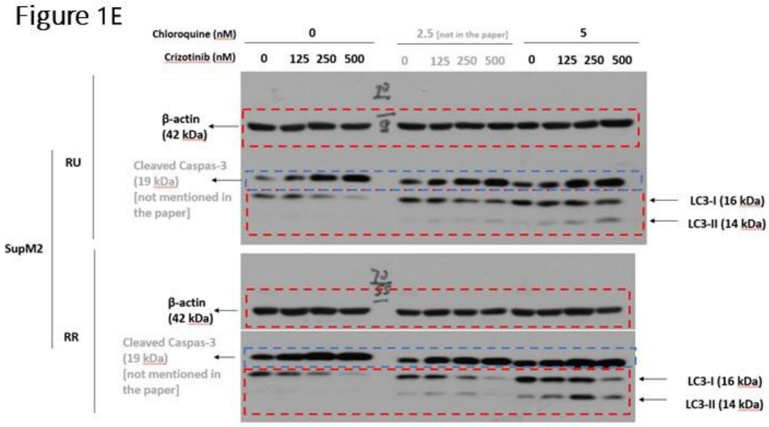

Figure 3

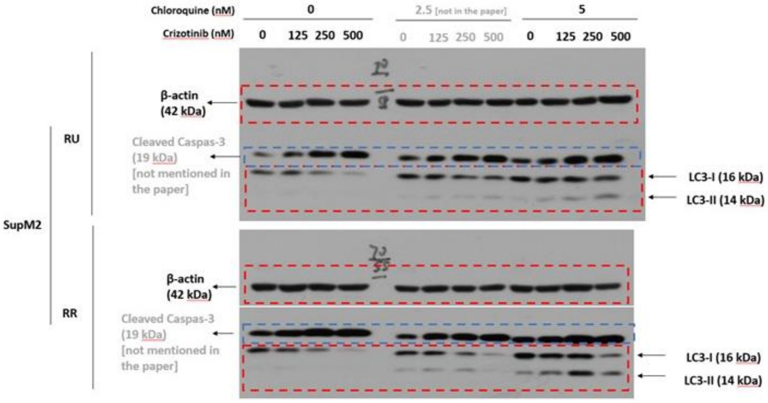

Figure 4

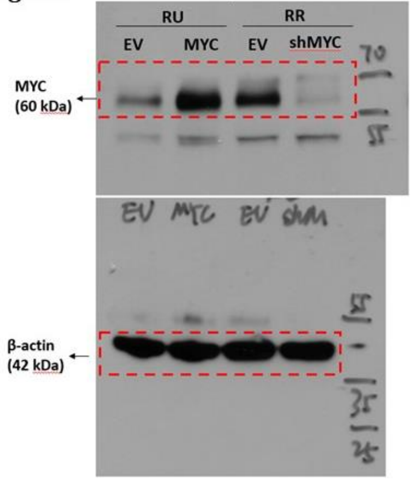

Figure S4. Original western blots of Figures 1, 3 and 4.
